# 

**Published:** 1915-07-17

**Authors:** 


### Gifts and Bequests.

Mr. William Balls, of Tynemouth, late head of the firm of Messrs. W. D. C. Balls and Son, shipowners, of North Shields, has left £1,000 to the Tynemouth Victoria Jubilee Infirmary.

Mr. Percy Ambrose Sewell Hickey, of Sackville Street, W., formerly of Stourbridge, Worcestershire, barrister, who left estate valued at £176,774 gross, has bequeathed £1,000 to Addenbrooke's Hospital, Cambridge.

Rates of Subscription: Twelve months, 6/6; Six months, 4/-; Three months, 2/-, post free to any address in the United Kingdom. Abroad, Twelve months, 8/8; Six months, 5/-; Three months, 2/6, post free.—Address The Subscription Dept., "The Hospital," The Hospital Building, 28/29 Southampton Street, Strand, London, W.C.

